# Magnetic Resonance Imaging-Based Radiological and Morphologic Analysis of the Pituitary Gland in the Setting of Non-Pituitary Intracranial Space-Occupying Lesions

**DOI:** 10.7759/cureus.115472

**Published:** 2026-08-30

**Authors:** Muhammad Kaleem, Muhammad Tahir Khan, Afra Ahmad, Hamza Manzoor, Haseeb Mehmood Qadri, Faiqa I Khan, Mahboob Ahmad

**Affiliations:** 1 Radiology, Woodlands Health Campus, Singapore, SGP; 2 Radiology, Lahore General Hospital, Lahore, PAK; 3 Clinical Radiology, Royal College of Radiologists, London, GBR; 4 Radiology, Punjab Institute of Neurosciences, Lahore, PAK; 5 Radiology, Chughtai Institute of Pathology, Lahore, PAK; 6 General Surgery, Lahore General Hospital, Lahore, PAK; 7 Neurological Surgery, Punjab Institute of Neurosciences, Lahore, PAK; 8 Neurosurgery, Punjab Institute of Neurosciences, Lahore, PAK

**Keywords:** brain, magnetic resonance imaging, morphology, morphometry, pakistan, pituitary gland, sella turcica

## Abstract

Background and objective

The pituitary gland is a small gland located in the pituitary fossa. Significant variations in pituitary gland size and shape exist across different age groups and genders. This variability can create uncertainty, making it challenging for radiologists to determine whether a certain size falls within a normal range. This study aimed to analyze the morphometric features of the pituitary gland in relation to age and sex among individuals without apparent pituitary disease or any medical condition affecting the pituitary gland.

Methodology

This retrospective observational study was conducted at the Department of Radiology, Punjab Institute of Neurosciences, Lahore, Pakistan. Clinical information and magnetic resonance imaging (MRI) reports of 502 consecutive eligible patients were retrieved between January 1, 2022, and December 31, 2024. Morphometric measurements of the pituitary gland in relation to age and sex were obtained from individuals with non-pituitary space-occupying lesions (SOLs) using T1-weighted midsagittal and T2-weighted coronal brain MRI images. The Mann-Whitney U test was performed to compare morphometric parameters between male and female participants, whereas the Kruskal-Wallis test was performed to assess differences across age groups. Post hoc pairwise comparisons with Bonferroni correction were subsequently performed to evaluate significant differences between age groups.

Results

With a cohort size of 502 and a slight male predominance, with 269 (53.58%) male participants, the majority of participants were in the 21-30-year age group, comprising 98 (19.5%) participants. A flat superior surface was observed in 245 (48.8%) participants. The mean pituitary height was 5.48 ± 1.49 mm and 5.32 ± 1.43 mm in female and male participants, respectively. The mean length was 9.45 ± 1.72 mm and 9.10 ± 1.60 mm in male and female participants, respectively. The mean pituitary width was 12.13 ± 4.7 mm and 11.8 ± 2.45 mm in male and female participants, respectively. The mean volume was 315.17 ± 137.11 mm³ and 314.89 ± 140.48 mm³ in male and female participants, respectively. Mean pituitary height was higher in female participants, whereas length, width, and volume were higher in male participants. However, only the difference in length was statistically significant (U = 27525.00, Z = −2.355, p = 0.019). The length of the gland showed a statistically significant linear increase across the age groups, from younger to older participants. Height, width, and volume displayed a peak-and-decline pattern, reaching a significant peak at age 21-30 years, then declining in older age.

Conclusion

The flat superior surface was the most commonly noted shape of the pituitary gland. The volume, width, and height of the pituitary gland increased gradually with age and declined in older adults. However, pituitary gland length increased with age, without any notable fluctuations in measurements across the age groups.

## Introduction

The pituitary gland is a small gland located in the sella turcica of the sphenoid bone. Significant differences exist among healthy individuals in pituitary gland size and shape, as well as in the depth of the sella [1]. As it regulates the activity of the majority of other hormone-secreting glands, it has been labeled the “master gland” [1,2]. Pregnant and nursing women have larger glands, whereas children and the elderly have smaller glands [2-5]. Age, sex, and even a person’s race can affect the size and morphology of this gland [1]. Because results might occasionally be very mild, a thorough evaluation of the pituitary gland is crucial [6,7].

Magnetic resonance imaging (MRI) is considered the preferred method for pituitary gland evaluation, as it offers higher resolution without the risks associated with ionizing radiation [2,8,9]. While it usually allows for a clear distinction between normal and abnormal sizes, there remains an area of overlap where radiologists face considerable difficulty in establishing whether a particular size is benign or pathological [1,10]. Accurate interpretation of pituitary dimensions necessitates a thorough understanding of its normative size, anatomical configuration, and physiological variations pertaining to the patient’s age and sex [6]. However, pituitary volume was not measured, and the patients included were ≤30 years.

A thorough search using PubMed Central and Google Scholar indicates a lack of studies, particularly from Pakistan, on pituitary morphology and size, including its anteroposterior, transverse, and craniocaudal dimensions and volume. This study aimed to highlight the morphometric analysis of the pituitary gland in relation to age and sex among individuals without apparent pituitary disease or any medical condition affecting the pituitary gland.

This article was previously posted to the Research Square preprint server on March 25, 2026.

## Materials and methods

This retrospective observational study was conducted at the Department of Radiology, Punjab Institute of Neurosciences, Lahore, Pakistan, involving data from patients who underwent MRI of the brain for non-pituitary intracranial space-occupying lesions (SOLs) between January 1, 2022, and December 31, 2024. The study was conducted in January and February 2025.

Inclusion and exclusion criteria

Individuals with non-pituitary intracranial SOLs aged up to 80 years, without midline shift (defined as a >5-mm shift of the septum pellucidum to one side), with a normal pituitary gland on brain MRI, and with no history of endocrine or systemic diseases or use of any medication that could influence the pituitary gland were included in the study. Individuals with pituitary abnormalities (e.g., tumors and cysts), a midline shift greater than 5 mm, a history of pituitary surgery or radiation, use of hormonal medications, or poor-quality MRI images were excluded.

Data collection and analysis

Patients’ medical records were scrutinized to determine the indication for noncontrast MRI and to ensure that they did not have medical conditions influencing pituitary size. Age was categorized to reduce potential age-related physiological variability bias when reporting the results. Additionally, patients with an empty sella or a sella filled with cerebrospinal fluid, and those with a pituitary gland height of less than 2 mm, were excluded from the study and subsequent analysis. Using the Picture Archiving and Communication System (PACS) workstation, four radiologists, each with five to seven years of relevant experience, independently carried out the morphometric measurements. The measurements were then examined, and disagreements were settled with discussion and agreement. In cases where ambiguity remained, the advice of a senior, experienced radiologist was sought to arrive at a final consensus measurement.

Brain MRI examinations included in this study had been performed as part of routine clinical care using a 3.0-Tesla Siemens MRI scanner. The standard noncontrast MRI protocol comprised T1-weighted midsagittal spin-echo and T2-weighted coronal spin-echo sequences. Coronal and sagittal images were selected with a matrix size of 320 × 256, a field of view (FOV) of 240 mm, and a slice thickness of 5 mm.

The pituitary gland’s length was measured on a midsagittal T1-weighted image as the maximum anteroposterior extent of the gland in the horizontal plane, as depicted in Figure 1. Its height was measured on the same sequence from the superior-most part to the inferior-most part of the pituitary gland. Its width was measured on a coronal T2-weighted image as the maximum transverse diameter of the pituitary gland. These measurements were made using the PACS measurement tools to avoid reporting bias. Four radiologists, each possessing five to seven years of experience, independently performed manual measurements on PACS, and the interobserver agreement among them was assessed.

**Figure 1:**
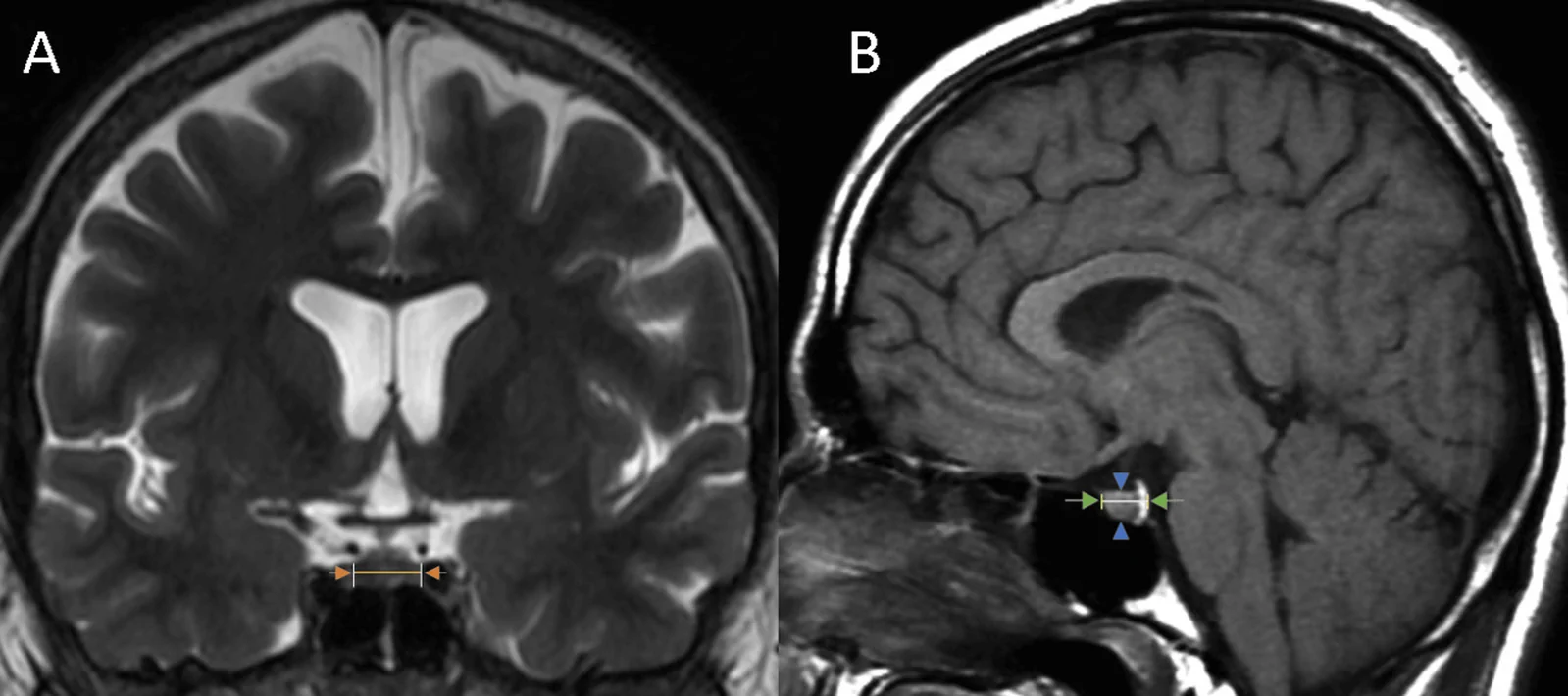
(A) Coronal T2-weighted image. The transverse dimension is measured from the right lateral part to the left lateral part of the pituitary gland, as indicated by the yellow line. (B) Midsagittal T1-weighted image. The anteroposterior (AP) dimension is measured from the anterior-most part of the anterior pituitary gland to the posterior-most part of the posterior pituitary gland, as indicated by the white line. The craniocaudal (CC) dimension is measured from the superior-most part to the inferior-most part of the pituitary gland, as indicated by the blue arrowheads.

Pituitary volume was calculated using the formula: volume = height × length × width × 0.52. This formula was derived from the sphere volume equation coefficient and cubic volume calculation: (4/3π)(r³)/(2r)³ = 3.1416/6 = 0.52 [4].

The shape of the pituitary gland was observed on the midsagittal T1-weighted image, categorizing the superior surface as flat, convex, or concave. All data were collected using Google Forms (Google Inc., USA). Measures of central tendency, frequency, and percentages were calculated using Microsoft Excel (Microsoft Corporation, Washington, United States). The Shapiro-Wilk test was utilized to assess the normality of the dataset. The Mann-Whitney U test was performed to examine the differences in morphometric parameters between the two sexes, whereas the Kruskal-Wallis test was performed to assess the differences across age groups. Subsequently, post hoc pairwise comparisons with Bonferroni correction were performed to evaluate significant differences between the different age groups.

The study was approved by the Institutional Review Board of Punjab Institute of Neurosciences on December 10, 2024, via Reference #1984/IRB/PINS/Approval/2024.

## Results

In this study, the sample size was 502, with a slight male predominance, with 269 (53.58%) male participants. The study analyzed pituitary gland dimensions (length, height, width, and volume) across 502 participants divided into eight age groups. The majority of participants were in the 21-30-year age group, comprising 98 (19.5%) participants, followed by the 31-40-year age group, comprising 84 (16.7%) participants, as shown in Table 1. The 5-9-year age group was the smallest among the age groups, with a total of 21 (4.2%) participants, as shown in Table 1.

**Table 1:** Age and gender-wise distribution of the studied cohort

Age group (years)	Male, n (%)	Female, n (%)	Total, n (%)
<5	21 (47.73%)	23 (52.27%)	44 (8.8%)
5–9	12 (57.14%)	9 (42.86%)	21 (4.2%)
10–14	17 (51.52%)	16 (48.48%)	33 (6.6%)
15–20	34 (51.52%)	32 (48.48%)	66 (13.1%)
21–30	48 (48.98%)	50 (51.02%)	98 (19.5%)
31–40	40 (47.62%)	44 (52.38%)	84 (16.7%)
41–50	27 (42.84%)	36 (58.14%)	63 (12.5%)
>50	70 (75.27%)	23 (24.73%)	93 (18.5%)

Regarding the morphology of the pituitary gland, the most common shape of the superior surface was flat, observed in 245 (48.8%) participants, as shown in Figure 2.

**Figure 2:**
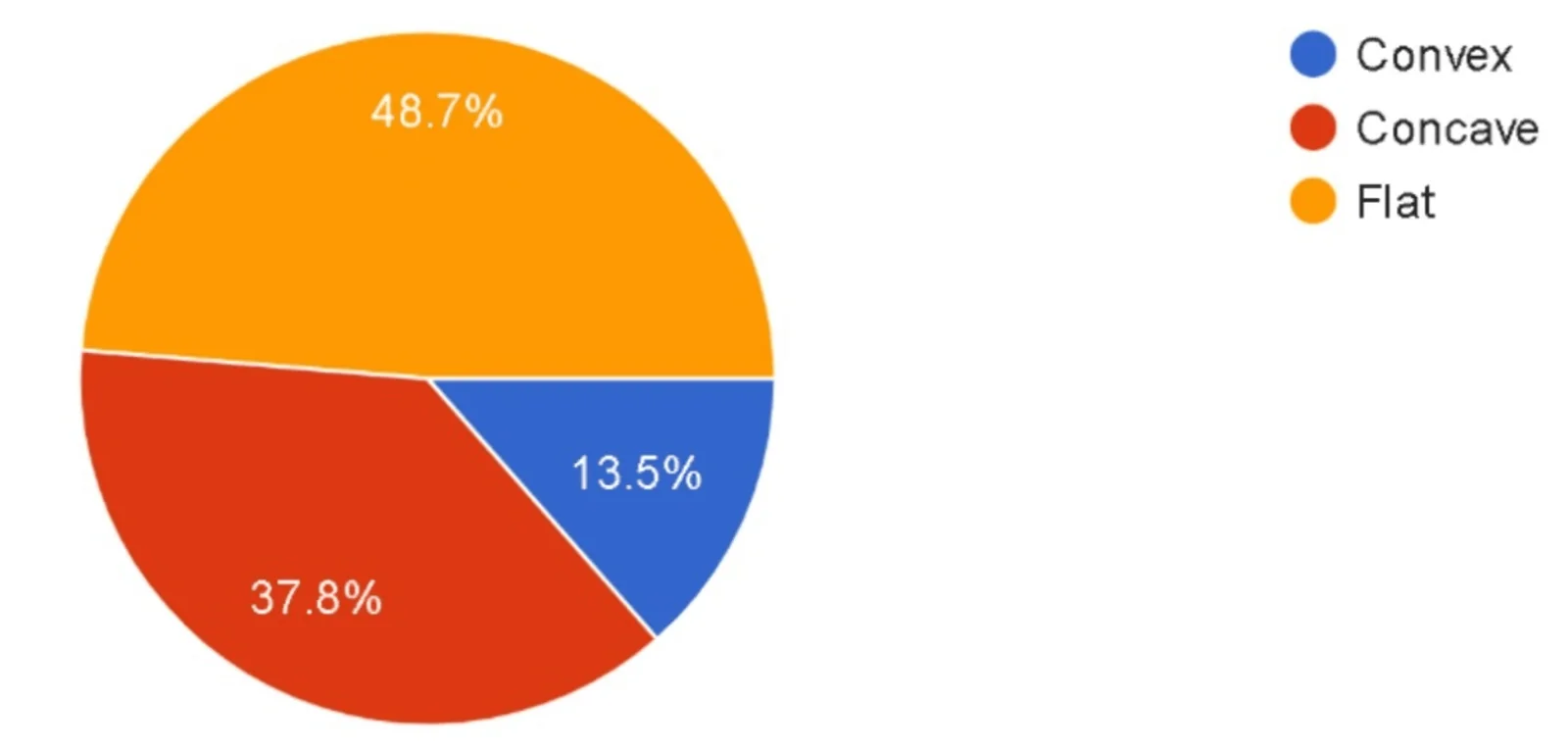
Pie chart showing three types of surfaces of pituitary gland

The mean pituitary height was 5.48 ± 1.49 mm and 5.32 ± 1.43 mm in female and male participants, respectively. The maximum mean height observed in the 15-20-year age group was 5.68 ± 1.20 mm in male participants and 6.17 ± 1.48 mm in female participants. Female participants showed higher pituitary height dimensions than male participants, as shown in Table 2. In the remaining age groups, male participants consistently exhibited larger pituitary gland dimensions than female participants, although the differences were relatively small.

**Table 2:** Mean pituitary gland height by gender and age group

Age group (years)	Male		Female	
	No. of subjects	Mean height (mm)	No. of subjects	Mean height (mm)
<5	21	3.66 ± 0.86	23	3.72 ± 0.71
5-9	12	4.41 ± 1.74	9	4.45 ± 0.70
10-14	17	5.38 ± 1.00	16	6.01 ± 1.32
15-20	34	5.68 ± 1.2	32	6.17 ± 1.48
21-30	48	5.68 ± 1.03	50	6.13 ± 1.4
31-40	40	5.13 ± 1.3	44	5.56 ±1.5
41-50	27	5.35 ± 1.2	36	4.93 ± 0.99
>50	70	5.64 ± 1.67	23	5.67 ± 1.97

The mean length was 9.45 ± 1.72 mm and 9.10 ± 1.60 mm in male and female participants, respectively. There was an overall increasing trend in length with age. However, no decline in the older age groups was observed. The maximum mean length was observed in the 41-50-year age group, i.e., 10.21 ± 1.67 mm in male participants and 9.64 ± 1.63 mm in female participants, with slightly higher values in male participants, as shown in Table 3.

**Table 3:** Mean pituitary gland length by gender and age groups

Age group (years)	Male		Female	
	No. of subjects	Mean length (mm)	No. of subjects	Mean length (mm)
<5	21	7.10 ± 1.67	23	7.14 ± 1.08
5-9	12	8.11 ± 1.67	09	7.77 ± 1.19
10-14	17	8.76 ± 1.77	16	8.36 ± 1.29
15-20	34	9.32 ± 1.67	32	9.15 ± 1.41
21-30	48	9.76 ± 1.68	50	9.379 ± 1.52
31-40	40	9.57 ± 1.62	44	9.623 ± 1.58
41-50	27	10.21 ± 1.67	36	9.64 ± 1.63
>50	70	10.10 ± 1.72	23	9.61 ± 1.67

The same pattern of increase in length dimension with age, followed by a decline in older age groups, was observed in the height dimension. Mean pituitary width was 12.13 ± 4.7 mm and 11.8 ± 2.45 mm in male and female participants, respectively. Pituitary gland dimensions increased with age, peaking in the 21-30-year age group for female participants, i.e., 13.01 ± 1.15 mm, and in the 31-40-year age group for male participants, i.e., 13.95 ± 1.18 mm, as shown in Table 4. Male participants exhibited higher pituitary width than female participants.

**Table 4:** Mean pituitary gland width by gender and age group

Age group (years)	Male		Female	
	No. of subjects	Mean width (mm)	No. of subjects	Mean width (mm)
<5	21	9.07 ± 1.73	23	9.11 ± 0.82
5-9	12	10.44 ± 0.89	9	9.78 ± 0.91
10-14	17	10.70 ± 0.95	16	11.9 ± 0.98
15-20	34	12.66 ± 1.02	32	12.71 ± 1.05
21-30	48	12.77 ± 1.12	50	13.01 ± 1.15
31-40	40	13.95 ± 1.18	44	12.1 ± 1.21
41-50	27	12.36 ± 1.23	36	12.14 ± 1.26
>50	70	11.87 ± 1.29	23	11.2 ± 1.32

The same pattern of increase in volume with age, followed by a decline in older age groups, was observed in the height dimension. The mean volume was 315.17 ± 137.11 mm³ and 314.89 ± 140.48 mm³ in male and female participants, respectively. The mean volume peaked at 372.20 ± 67.89 mm³ in male participants in the 21-30-year age group and at 387.24 ± 66.78 mm³ in female participants in the same age group, as shown in Table 5. Regarding children, including those younger than 5 years and those aged 5-9 years, collectively 65 (13%), all pituitary gland parameters gradually increased as age advanced. A gender difference was also observed.

**Table 5:** Mean pituitary gland volume by gender and age group

Age group (years)	Male		Female	
	No. of subjects	Mean volume (mm^3^)	No. of subjects	Mean volume (mm^3^)
<5	21	123.94 ± 25.34	23	126.48 ± 26.12
5-9	12	219.31 ± 34.56	9	176.34 ± 33.45
10-14	17	267.45 ± 45.67	16	326.59 ± 44.56
15-20	34	344.67 ± 56.78	32	346.45 ± 55.67
21-30	48	372.20 ± 67.89	50	387.24 ± 66.78
31-40	40	301.76 ± 72.34	44	340.76 ± 71.23
41-50	27	359.5 ± 75.67	36	306.81 ± 74.56
>50	70	337.24 ± 78.89	23	310.83 ± 77.78

The Mann-Whitney U test was performed to assess statistically significant differences in morphometric parameters between both genders. The length of the pituitary gland showed a statistically significant increase in male participants compared with female participants (U = 27525.00, Z = −2.355, p = 0.019). The other parameters, including width (U = 30559.00, p = 0.630), height (U = 29983.00, p = 0.403), and volume (U = 30956.50, p = 0.814), did not show any statistically significant differences (Table 6).

**Table 6:** Mann Whitney U test for gender-based comparison of morphometric parameters

Variable	U	Z	p-value
Length	27525.0	-2.355	0.019
Width	30559.0	-0.481	0.630
Height	29983.0	-0.836	0.403
Volume	30956.5	-0.236	0.814

The Kruskal-Wallis H test was employed to assess significant differences in morphometric parameters across the eight age groups. Statistically significant differences were observed among the age groups for all variables (Table 7).

**Table 7:** Kruskal–Wallis test for the age-group comparison of morphometric parameters

Variable	df	H (χ²)	p-value
Length	7	100.486	<0.001
Width	7	84.692	<0.001
Height	7	105.793	<0.001
Volume	7	134.416	<0.001

Post hoc pairwise comparisons with Dunn-Bonferroni correction were performed to analyze which age groups had significant differences. An age-dependent increase in pituitary morphometric parameters was observed, particularly in gland length and volume, representing enlargement with increasing age (Table 8).

**Table 8:** Post hoc pairwise comparison among age groups with Dunn-Bonferroni correction

Parameter and age group	p-values
Length	
>50 vs <5 years	0.001
>50 vs 5-9 years	0.0013
>50 vs 10-14 years	0.0015
21-30 vs <5 years	0.001
Width	
>50 vs 21-30 years	0.006
>50 vs <5 years	0.001
Height	
>50 vs <5 years	0.001
>50 vs 5-9 years	0.012
41-50 vs 21-30 years	0.00085
41-50 vs 15-20 years	0.019
Volume	
>50 vs <5 years	0.001
>50 vs 5-9 years	0.0003

## Discussion

Based on a literature search using PubMed and Google Scholar, to the best of our knowledge, this is one of the largest systematically conducted studies in the Pakistani population to obtain morphometric measurements of the pituitary gland, encompassing individuals of all ages and sexes without apparent radiological abnormality or compressive involvement of the pituitary gland in patients with non-pituitary intracranial SOLs.

The shape and measured dimensions of the pituitary gland in the Pakistani cohort are consistent with measurements documented in the global literature. A flat superior surface was the most commonly noted shape of the pituitary gland. The height of the gland was higher in female participants, whereas length, width, and volume were higher in male participants. The volume, width, and height of the pituitary gland increased gradually with age and declined in older adults. However, a linear relationship was noted between length and increasing age; no decline in the older age groups was observed.

MRI is the preferred examination for visualizing the pituitary gland, and the radiologist must be aware of the normal size and volume of the gland and the variations related to age and sex, as small changes in the size of the gland could have a considerable effect on other neuroendocrine glands. Borderline pituitary abnormalities are frequently encountered, such as small microadenomas, physiological alterations of the gland, and inflammatory diseases, but there is a constant dilemma for radiologists in reporting such cases. Although there is a substantial body of literature on the normal size and variations of the gland from other countries, such literature is scarce in Pakistan. This study was designed to recognize the morphometric measurements observed in a clinical cohort of non-pituitary intracranial SOLs and their variations with age and sex in the Pakistani population [1,2].

The pituitary gland is a pea-sized gland that sits in the pituitary fossa of the sphenoid bone and is surrounded by the sella turcica, covered by the dural fold called the diaphragma sellae. In humans, it comprises two main lobes, anterior and posterior, with the intermediate lobe joining them together [1]. The most common shape of the superior surface of the pituitary gland was flat, followed by a concave surface. According to a study conducted on the Indian population, the most common superior surface shape observed was flat, followed by convex and concave [11]. Another study conducted on the Nepali population with a sample size of 137 showed similar results [2]. According to a study by Singh et al., a concave superior surface was the most prevalent pituitary gland morphology [8].

In 2023, a meta-analysis of pituitary morphology was conducted. This analysis comprised a total of 72 studies. A convex superior surface of the pituitary glands was found to be the most prevalent [1]. A similar outcome was noted in a 2008 local study [6].

We found that the mean pituitary height differed between male and female participants; however, the difference was not statistically significant. In our study, the mean height of the pituitary glands in male and female participants was 5.32 ± 1.43 mm and 5.48 ± 1.49 mm, respectively. The maximum mean height observed in the 15-20-year age group was 5.68 ± 0.78 mm for male participants and 6.17 ± 0.74 mm for female participants in the same age group. According to Singh et al., the maximum mean pituitary height was observed earlier, i.e., in the 10-14-year female age group [8]. Female participants showed higher pituitary height dimensions than male participants. Similar findings were observed in another study conducted by Ikram et al. [6]. Another study by Singh et al. showed higher pituitary height dimensions (5.80 ± 1.32 mm and 5.37 ± 1.25 mm in female and male participants, respectively) compared with our study. In another study conducted by Suzuki et al., pituitary height dimensions were lower (5.0 ± 1.7 mm in women and 4.7 ± 1.4 mm in men) than in our study [7]. In addition to these two studies, a large number of other investigations have found that women have larger pituitaries than men [2,6]. Suzuki et al., Elster et al., and Denk et al. reported peak pituitary height in the second decade of life and maximum height of the pituitary gland in the third decade of life [7,12,13]. Denk et al. reported mean pituitary heights of 6.1 ± 0.1 mm for female participants and 5.6 ± 0.2 mm for male participants, which were higher than those in our study [13].

Pituitary height in both sexes showed a trend of steady decline with age after peaking in the second decade of life. In female participants, we observed that the mean pituitary height increased once again throughout the fifth decade of life. Previously, Singh et al. also noted that older female participants had higher pituitary gland heights [1,8,12]. They ascribed the phenomenon of increased pituitary height in elderly females to greater gonadotroph activity as a result of the loss of negative feedback. Both male and female participants in our study showed an increase in pituitary height after the age of 50 years. Previous research has also noted this observation [2,8].

According to earlier research, these morphological alterations in the pituitary may be caused by variations in hormone levels. Luteinizing hormone (LH) production during puberty may be linked to the increase in pituitary height during this period of growth [1,14]. Additionally, the physiological changes in neuroendocrine hormones between younger and older subjects may be the cause of the comparatively higher pituitary height in young patients, both male and female. Age-related changes in endocrine state and pituitary gland physiological atrophy are also demonstrated by the decline in pituitary height [1,14].

Pituitary height increases did not correspond with changes in the pituitary gland length. There was no discernible spurt in the pituitary gland length. There was still a discernible trend in the differences in mean pituitary length and width between the sexes. Pituitary length and width are not very useful in reflecting total pituitary size, according to previous authors [7,8]. This is due to the pituitary glands’ inferior and lateral bony boundaries. Pituitary height changes in response to changes in pituitary size, but neither length nor width changes. Pituitary height is the most straightforward and accurate measure of pituitary size due to the unique structure of the pituitary gland [4].

In our study, age- and sex-specific changes in the calculated pituitary gland volume closely matched changes in pituitary gland height. Similar findings were observed in other studies as well [3,4,8]. One study conducted on prepubertal children aged under 10 years used 3D MRI data for pituitary gland volume. The study stated that the volume obtained showed a linear relation with age but a weak relation with height and other parameters [4]. According to this study, volume assessment by the 3D method is robust compared with manual methods [4]. In both sexes, pituitary volume expanded gradually from the first year onward and peaked in the third decade. In our study, the difference in pituitary volumes between male and female participants in the 21-30-year age group was not statistically significant. In one study, pituitary volume increased gradually, and a surge was observed in the second half of the second decade, similar to our study [8,12,13]. The basal serum concentrations of gonadotropic hormones such as LH and follicle-stimulating hormone (FSH) decreased after puberty up to the fifth decade of life [14]. However, in female participants, the concentrations of these hormones start to increase in their fifth and sixth decades because of an age-related decrease in circulating gonadal steroid hormones and an increase in gonadotropin-releasing hormones [1]. According to our study's findings, women in the same age group have larger pituitary gland height (i.e., 50 years and above). Prior research has also demonstrated that older adults' pituitary height increases, potentially as a result of compensatory hypertrophy following a marked reduction in the gonadal steroid feedback effect [1,5,14]. Mohieldin et al. noted a significant correlation between pituitary gland height in both sexes during the age ranges of 11-21 years, 22-32 years, 33-43 years, and 44-54 years, respectively [15].

Different authors have reported different mean pituitary heights for different groups. Using data from other groups as a benchmark for the Pakistani population could not be considered rational. A local study evaluated normal pituitary height in relation to age and sex in the Pakistani population [6]. The study included 220 patients aged ≤30 years; no other pituitary features such as length, width, or volume were assessed. However, children under five years of age and individuals older than 30 years were not included. Additionally, the limitations of Singh et al.'s research on pediatric groups were discussed earlier [8].

Our study provides MRI-based morphometric reference data for pituitary gland dimensions within a clinical cohort of patients undergoing evaluation for non-pituitary intracranial lesions.

Limitations

Age and gender blindness could not be ensured during the data acquisition process because the PACS system was used. Moreover, as the data were collected retrospectively, the study is limited by its ability to account for other potential confounding variables, such as body mass index (BMI). The data were obtained from a single hospital. Another limitation was the inclusion of patients with suspected or confirmed illnesses rather than healthy volunteers, as we cannot afford to order expensive neuroimaging in human beings where there is no symptomatic or diagnostic indication; hence, this might not reflect the normal population. The MRI slices were 5 mm apart, which might not reflect accurate measurements in such a small structure. The radiological characteristics of intracranial lesions were not taken into account in this study. The grouping of female participants aged 15-20 and 21-30 years without hormonal adjustment is a limitation of our study. The formal evaluation of measurement reproducibility is limited by the lack of quantitative observer-agreement statistics, such as an intraclass correlation coefficient, even though measurements were taken by four skilled radiologists and disagreements were settled by consensus, consulting senior experts when needed.

Future prospective studies should incorporate hormonal assays and menstrual cycle phase documentation to better isolate age-related from hormone-related changes. The inclusion of participants aged 51-80 years within a single age group may have masked important age-related differences in pituitary gland morphology, particularly those related to physiological involution, postmenopausal hormonal changes, and advanced aging. Additionally, our study included fewer pediatric patients. The 3D volume approach was not used in our study.

## Conclusions

The flat superior surface was the most commonly noted shape of the pituitary gland. The height of the gland was higher in female participants, whereas length, width, and volume were higher in male participants. The volume, width, and height of the pituitary gland showed an age-related pattern, peaking in early adulthood followed by a decline in older adults. However, pituitary gland length increased with age, with no fluctuations observed across the age groups. Our findings represent pituitary morphometric measurements in patients with non-pituitary intracranial lesions without apparent compressive effects on the pituitary, rather than pure normative values.

## References

[1] Bonczar M, Wysiadecki G, Ostrowski P (2023). The morphology of the pituitary gland: a meta-analysis with implications for diagnostic imaging. Brain Sci.

[2] Jha A, Paudel N, Nepal S (2020). Normal pituitary gland size and morphology and its variations related to age and gender: an MRI evaluation. J Nepalgunj Med Coll.

[3] Çolaklar A, Fitoz ÖS (2023). Pituitary gland volumes in children with normal endocrine function. Pediatr Radiol.

[4] Marziali S, Gaudiello F, Bozzao A (2004). Evaluation of anterior pituitary gland volume in childhood using three-dimensional MRI. Pediatr Radiol.

[5] Berntsen EM, Haukedal MD, Håberg AK (2021). Normative data for pituitary size and volume in the general population between 50 and 66 years. Pituitary.

[6] Ikram MF, Sajjad Z, Shokh I, Omair A (2008). Pituitary height on magnetic resonance imaging observation of age and sex related changes. J Pak Med Assoc.

[7] Suzuki M, Takashima T, Kadoya M (1990). Height of normal pituitary gland on MR imaging: age and sex differentiation. J Comput Assist Tomogr.

[8] Singh AK, Kandasamy D, Garg A, Jyotsna VP, Khadgawat R (2018). Study of pituitary morphometry using MRI in Indian subjects. Indian J Endocrinol Metab.

[9] Whittle S, Barendse M, Pozzi E, Vijayakumar N, Simmons JG (2020). Pubertal hormones predict sex-specific trajectories of pituitary gland volume during the transition from childhood to adolescence. Neuroimage.

[10] Kuo M, Maya MM, Bonert V, Melmed S (2021). Prospective evaluation of incidental pituitary imaging findings in the sella turcica. J Endocr Soc.

[11] Krishna GK, Bopaiah H, Sakalecha AK (2024). Morphometric analysis of pituitary gland using magnetic resonance imaging in Indian subjects. Asian J Med Sci.

[12] Elster AD, Sanders TG, Vines FS, Chen MY (1991). Size and shape of the pituitary gland during pregnancy and post partum: measurement with MR imaging. Radiology.

[13] Denk CC, Onderoğlu S, Ilgi S, Gürcan F (1999). Height of normal pituitary gland on MRI: differences between age groups and sexes. Okajimas Folia Anat Jpn.

[14] Yadav P, Singhal S, Chauhan S (2017). MRI evaluation of size and shape of normal pituitary gland: age and sex related changes. J Clin Diagn Res.

[15] Mohieldin AE, Mohamed DA, Mahmoud MZ (2016). Effect of age and gender variation in normal pituitary gland height using magnetic resonance imaging. J. Adv. Med. Med. Res.

